# Automatic generation of bioinformatics tools for predicting protein–ligand binding sites

**DOI:** 10.1093/bioinformatics/btv593

**Published:** 2015-11-05

**Authors:** Yusuke Komiyama, Masaki Banno, Kokoro Ueki, Gul Saad, Kentaro Shimizu

**Affiliations:** ^1^Human Genome Center, The Institute of Medical Science, The University of Tokyo, Minato-ku, Tokyo 108-8639, Japan and; ^2^Department of Biotechnology, Graduate School of Agricultural and Life Sciences, The University of Tokyo, Bunkyo-ku, Tokyo 113-8657, Japan

## Abstract

**Motivation:** Predictive tools that model protein–ligand binding on demand are needed to promote ligand research in an innovative drug-design environment. However, it takes considerable time and effort to develop predictive tools that can be applied to individual ligands. An automated production pipeline that can rapidly and efficiently develop user-friendly protein–ligand binding predictive tools would be useful.

**Results:** We developed a system for automatically generating protein–ligand binding predictions. Implementation of this system in a pipeline of Semantic Web technique-based web tools will allow users to specify a ligand and receive the tool within 0.5–1 day. We demonstrated high prediction accuracy for three machine learning algorithms and eight ligands.

**Availability and implementation:** The source code and web application are freely available for download at http://utprot.net. They are implemented in Python and supported on Linux.

**Contact:** shimizu@bi.a.u-tokyo.ac.jp

**Supplementary information:** Supplementary data are available at *Bioinformatics* online.

## 1 Introduction

The identification of protein–ligand binding sites is important for understanding protein function. Many bioinformatics methods have been proposed for ligand-binding site identification or prediction. Computational methods are useful because they can be applied rapidly and at low cost, compared with biochemical experiments. They have a wide range of applications in enzyme design, drug discovery, chemical genetics, and other fields.

The ligand-binding site prediction methods can be classified into sequence-based and structure-based methods. With high-throughput sequencing technologies yielding large amounts of sequence data, sequence-based methods can be applied to a wide range of proteins, even those whose structures have not been determined, and can be used for genome-wide analysis. In this study, we focused on sequence-based methods.

When the structure of a target protein is known, structure-based methods can achieve high accuracy. However, if modeled structures are used, additional computation time is required and accuracy depends on model quality. The Critical Assessment of Protein Structure Prediction (CASP) (Moult *et al.*, 2014) and the Continuous Automated Model EvaluatiOn (CAMEO) (Haas *et al.*, 2013) are world-wide experiments for protein structure prediction, and they have the category of ligand-binding site prediction. Many structure-based or model-based prediction methods have been assessed in this category. Some structure-based methods use structure similarity to template libraries. COFACTOR threads the structure through the BioLiP protein function database using local and global structure matches to identify functional sites and homologies (Roy *et al.*, 2012). COACH uses both structures and sequences; it recognizes ligand-binding templates from BioLiP by binding-specific substructure and profile comparisons (Edgar and Sjölander, 2004; Yang *et al.*, 2013a). It also provides a meta-server that combines the results with those from other methods including COFACTOR, FINDSITE and ConCavity to generate final predictions (Brylinski and Skolnick, 2009; Roy *et al.*, 2012; Yang *et al.*, 2013b).

Among sequence-based methods, LIBRUS (Kauffman and Karypis, 2009) is a profile-based ligand-binding site prediction method that uses both sequence homology and machine learning. LigandRFs uses a random forest (RF) ensemble to identify ligand-binding residues from sequence information alone (Chen *et al.*, 2014). The performance comparison with these methods is described here. There are also prediction methods that have been developed for specific ligands. However, a binding-site prediction tool for a user-specified ligand is not always available. It will be convenient if a system can predict binding sites for any ligand specified by the user.

Here, we describe a system for automatically generating a tool to predict the binding site for any ligand. This system takes as inputs a target ligand and an amino acid sequence to which the ligand binds, and as a prediction framework it constructs homology profiles to gather information on homologous proteins, using machine learning algorithms. This framework can be applied in common to numerous ligand types. The generated tools may be customized for individual ligand types to further improve prediction accuracy. The system can select and use three machine learning algorithms: support vector machine (SVM), neural network (NN) and RF, and can perform automatic optimization of the parameters of each algorithm. Its framework is based on semantic network technology and can be further extended to incorporate various features of proteins. For example, protein structural information can be used and other machine learning algorithms can be easily added to our framework.

The basic procedures of machine learning-based protein–ligand binding site prediction involve (i) construction of training datasets, (ii) extraction of sequence features and (iii) machine learning performance and parameter optimization. In our system, these steps are executed automatically as a pipeline in the Web application the University of Tokyo Proteins (UTProt) Galaxy (Supplementary Note S1). These steps produce predictors of ligand-binding sites for ligands of interest. The predictors generated by the system are displayed in the common user interface and can be used in the same manner. The system also stores these predictors in the pipeline workflow for subsequent use.

Using the structures of protein–ligand interaction sites extracted from Protein Data Bank (PDB) and the annotated sequences from Universal Protein Resource (UniProt) (Estrada *et al.*, 2012; Magrane and Consortium, 2011), we developed the Protein–Ligand Binding Site Pair Residue (PLBSP Residue) database. As a component of the pipeline system, this system collects positive (binding sites) and negative (non-binding sites) datasets for any specified ligand.

UTprot Galaxy employs the Galaxy framework, which is a customizable pipeline of open-source Web applications (Blankenberg *et al.*, 2010). Additional functional modules can be added to the present system and the developed program can be easily integrated, as a module, into other programs. Users can conduct the pipeline using a generic browser and graphical user interface; the framework also supports the graph database based on Semantic Web technology. Semantic Web technology is used as a method of linking the existing databases PDB (Kinjo *et al.*, 2012) and UniProt with the newly developed resource description framework (RDF) databases PLBSP Residue and RDF SIFTS that contain in-house data. The European Bioinformatics Institute (EBI) developed the structure integration with function, taxonomy and sequences (SIFTS) platform as an up-to-date resource for residue-level mapping between UniProt and PDB entries (Velankar *et al.*, 2013). RDF is a core technology of the Semantic Web in the graph database. RDF represents graph data and regulates metadata format by the World Wide Web Consortium. In graph theory, a directed graph has three components: two nodes and a directed edge. A semantic graph assigns, as a RDF representation, a subject to one node, an object to another node, and a predicate to the edge. Each graph component expresses data as uniform resource identifiers (URIs) using ontology terms. This common form is useful for database integration, making it easy to express data relationships and link data that differ in underlying schema. The SPARQL Protocol and RDF Query Language (SPARQL) can operate a graph database that loads a dataset of RDF (Belleau *et al.*, 2008; Katayama *et al.*, 2014; Tanaka *et al.*, 2014; Willighagen *et al.*, 2013; Wimalaratne *et al.*, 2015). The fitting of data to the RDF facilitated the development of the system and the automatic generation of prediction tools.

## 2 Methods

**Fig. 1.**
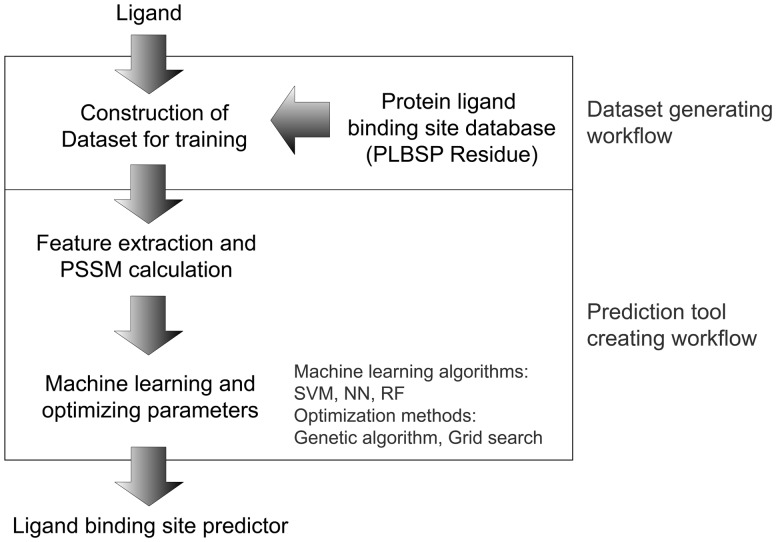
The pipeline workflow for automatic generation of ligand-binding site predictive tools. A user specifies the name of the ligand (chemical compound) for which a binding-site prediction tool is desired. The pipeline constructs the dataset for training, extracts the sequence features, and automatically performs machine learning and parameter tuning. As the backend of the pipeline system, we developed the PLBSP Residue database based on Semantic Web (linked open data) technologies. The pipeline output is a protein–ligand-binding prediction tool

The present pipeline system, UTProt Galaxy, comprises a dataset-generating workflow and a prediction tool-creating workflow (Fig. 1).

### 2.1 Dataset-generating workflow

We analyzed all PDB files of X-ray crystal structure for extracting protein–ligand binding site using an octree data structure. The octree is used because it permits easy and efficient searching and comparison of structures. We calculated the atomic distance between a residue and ligand at the binding site. In this study, we defined ligand-binding residues as those that contain at least one atom within *n* Å of any ligand atom in an experimentally determined structure. The distance *n* was set at 3.5–6 and 5 Å was used as the default value. Ligands connected by covalent bonds were excluded, and then information describing covalent ligand–ligand and ligand–residue bonds was extracted from the PDB file.

We developed the PLBSP Residue graph database of ligand-binding residues for automatic generation of datasets for machine learning. PLBSP Residue is based on Semantic Web (linked open data) technologies; we modeled the graph database schema of PLBSP in RDF for analyzed in-house data. This database was constructed from all ligand-binding protein structures in the PDB. We used a public RDF dataset and ontology from PDB, PDB Chemical Component Dictionary (CCD) and UniProt with in-house data for the annotation. PDB sequences were mapped to UniProt to link multiple PDB sequences to a sequence, to obtain full protein sequences, and to link to various information. Other information linked to UniProt can be referenced. Ligand information from ChEBI, UniChem and SIFTS was also incorporated into PLBSP Residue. Using Semantic Web technologies, we can link various information sources and integrate system components dynamically (Supplementary Note S2).

**Fig. 2.**
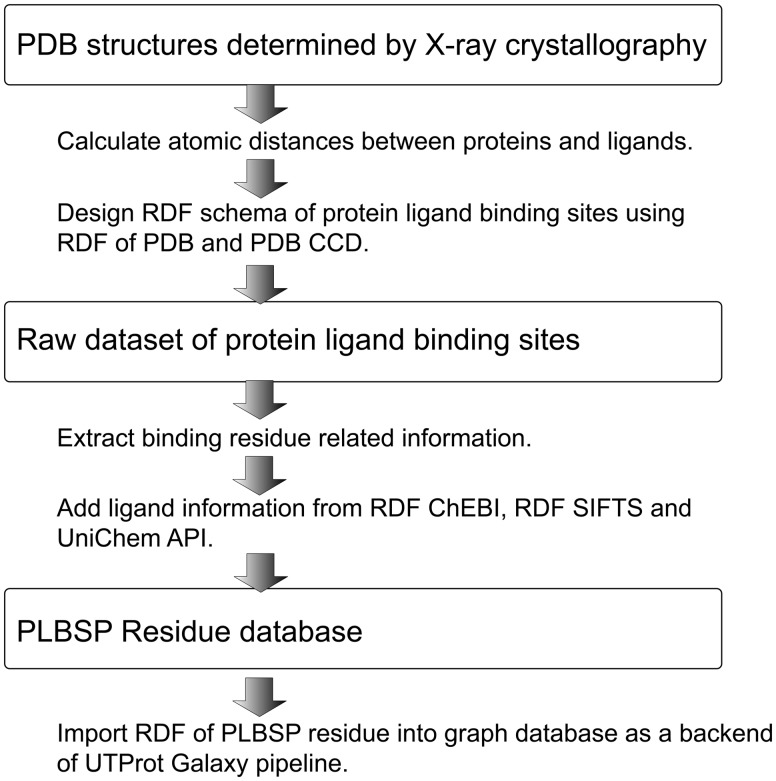
Construction process of PLBSP Residue database. We use ligand-binding PDB structures determined by X-ray crystallography. Ligand-binding residues are defined as those that contain at least one atom within *n* Å of any ligand atom. The atomic distance is calculated for all pairs of ligand atoms and ligand-binding residue atoms. Ligands connected by covalent bonds are excluded. We modeled the graph database schema of PLBSP in RDF using a public RDF dataset and ontology from PDB, PDB CCD and UniProt. Ligand information is added from RDF ChEBI, RDF SIFTS and UniChem API

PDB contains protein molecule structures that were determined under various experimental conditions. It also contains partially determined structures. For machine learning, protein sequences and structural relationships should be handled consistently. Correspondences between residue numbers in PDB and those in UniProt were mapped as RDF by converting models from spreadsheets to graph structures based on information from EBI SIFTS, which describes cross-reference information for protein-related databases such as PDB and UniProt. Using the RDF database of PLBSP Residue, it became possible to use complete sequence data that match one or more PDB entries and to perform cross-reference analyses of UniProt- or PDB-related databases and the current ontology release on the Web (Fig. 2).

UTProt Galaxy extracts IDs from PDB CCD for specified ligands. PDB CCD ID lists that were extracted from the PLBSP Residue database were classified using compound ontology chemical entities of biological interest (ChEBI). For example, the pipeline uses a PLBSP Residue database and performs a reasoning search using the following ontology vocabularies: urine nucleotide (ChEBI ID:26395), lipid (ChEBI ID:18059) and iron cation (ChEBI ID:24875). The pipeline then obtains a correspondence list mapped to ChEBI ID from the PDB CCD three-character ID (the same ID as the HETATM record in the PDB file) using the application programming interface of UniChem, which is a ‘unified chemical structure cross referencing and identifier tracking system’ (Chambers *et al.*, 2013). The reasoning search permits a ligand group search based on ChEBI ontology hierarchies. The specified ligand and ligands at lower ontology layers are selected as a target ligand group that partially takes into account the similarity among ligands. In this study, the pipeline generated lists of non-redundant ligand-binding amino acid sequences that corresponded to ligand groups and ligand-binding residues using the dataset-generating workflow.

The dataset-generating workflow involves ligand-binding protein acquisition followed by the removal of sequence redundancy and the collection of ligand-binding residues. In the ligand-binding protein acquisition step, proteins that bind the specified ligand are identified by searching the PLBSP Residue database and amino acid sequences are collected. The removal of sequence redundancy uses CD-HIT (Fu *et al.*, 2012), which removes redundancy from amino acid and DNA sequences. The default condition for removing redundancy is 90% identity and 50% coverage of sequences. During the step of collection of ligand-binding residues, the protein and ligand type are specified, and then the list of best-suited ligand-binding residues is obtained.

### 2.2 Prediction tool-creating workflow

The pipeline system generates protein–ligand binding prediction tools that predict whether or not each residue in a protein is a part of a ligand-binding site (a ligand-binding residue). In the current design, three machine learning algorithms: SVM, NN and RF can be used for prediction. We employed the Python-based machine learning packages scikit-learn and LIBSVM (SVM), PyBrain (NN) and RandomForestClassifier (RF), We also used NumPy and SciPy as numerical computing packages in Python (Breiman, 2001; Chang and Lin, 2011; Pedregosa *et al.*, 2011; Schaul *et al.*, 2010).

The positive training dataset is the one produced in the dataset-generating workflow. The negative dataset comprises residues within 5–25 residues of each ligand-binding residue. Position-Specific Iterated BLAST (PSI-BLAST) was used to construct multiple sequence alignments of homologous proteins to generate homology profiles for each sequence in the positive dataset, on the basis of the amino acid frequencies at each alignment position (Altschul and Koonin, 1998). The feature vector, which was taken as an input to the machine learning algorithms, was generated from the homology profile. In PSI-BLAST, two iterations were performed using the NCBI non-redundant (nr) protein database (Pruitt *et al.*, 2005).

**Fig. 3.**
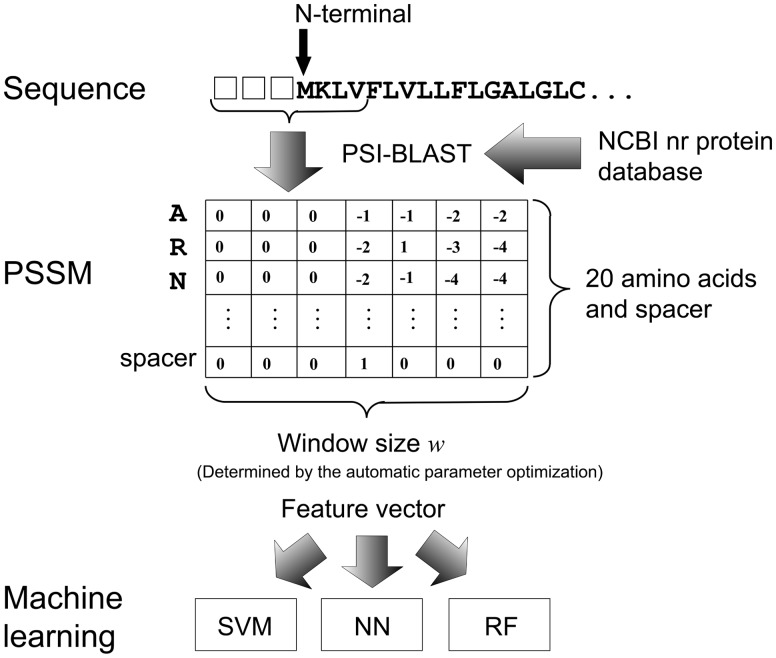
Feature extraction and machine learning. We generate a PSSM profile by performing two iterations of PSI-BLAST using the NCBI non-redundant (nr) protein database. The feature vector is a PSSM profile of *w* consecutive residues and the center residue is the target residue for which the prediction is being made. The dimension of the feature vector is w × 21, which corresponds to the number of amino acids (20 plus an N- or C-terminal spacer). The feature vector can be taken as input to the three machine learning algorithms

The current system uses a sliding-window approach in which the feature vector is a position-specific scoring matrix (PSSM) profile of *w* consecutive residues and the center residue is the target residue for which the prediction is being made. The dimension of the feature vector is *w* × 21, which corresponds to the number of amino acids (20 plus an N- or C-terminal spacer). PSSM profile is then converted into a feature vector with window size *w*. A spacer is a feature that indicates whether or not the site is beyond the N or C terminus. When the target residue is at the terminus of the sequence (either the C or the N terminus), it is assigned a value of 1. For all other residues in the window, the terminal spacer is set to 0 (Fig. 3).

Our system enables using the residue conservation in a multiple sequence alignment of homologous proteins as another feature of machine learning. Because the effectiveness of this option depends on the types of ligands, we did not use this option for performance evaluation in this study. Other features such as various properties of amino acids, including charge, hydrophobicity, size, etc., can be conceived. These properties can be more effective than PSSM profile when some specific interactions exist between the proteins and the ligands, but we used PSSM profile here because it can represent more general properties of binding sites.

This system automatically optimizes the parameters of machine learning: the optimal cost and Gaussian kernel parameters in SVM; the number of neurons and the learning rate in NN; the numbers of trees and of tries per tree in RF; and the window size *w* in all the machine learning algorithms. For parameter optimization, the system can use the genetic algorithm (GA) and grid search.

The conditions used by the GA are as follows: the tournament selection method, 100 generations, 20 individuals, a point mutation rate of 0.05, the single-point crossover method, a crossover rate of 0.8, a gene initialized from a continuous or discrete uniform distribution. The genotype was then converted into a fitness function using the following procedure. The grid search exhaustively generates candidates from a grid of parameter values.

The system uses cross-validation for parameter optimization. The results of the cross validation are evaluated using the fitness score AUC. The parameters obtained in each generation of GA step or each step of grid search are stored in a SQLite database. If the resulting parameters are the same as those of the previous generation, the calculation is removed using a cache technique. This practice increases the speed of calculation and reduces the total pipeline processing time.

### 2.3 Computational environment

The computational experiments were run within the following cloud environment of a virtual machine powered by Amazon Web Service (AWS): instance type, m1.xlarge for general computing use; OS, Ubuntu 12.04.5 LTS (64 bit); Linux kernel, (GNU/Linux 3.2.0-77-virtual x86_64); CPU, 8 ECU Intel(R) Xeon(R) CPU E5645 @ 2.40 GHz) × 4 vCPUs; main memory, 15 GB; storage, 420 GB × 4; EBS optimization, available; and network performance, high. GA and grid search were tested on a standalone Windows 8 machine with CPU AMD FX-8350 and 32 GB main memory running R (3.2.1-64 bit) and RStudio (v0.89.501).

## 3 Results and discussion

### 3.1 Performance of automatically generated predictors

We used our pipeline system to generate predictors for purine nucleotide, lipid, iron cation (Fe), zinc cation (Zn), manganese cation (Mn), α-glucose, flavin adenine dinucleotide (FAD), adenosine monophosphate (AMP) and iron/sulphur cluster (SF4) binding sites. Accordingly, the dataset summary in Table 1 includes the number of ligand types, the number of interacting proteins and the number of ligand-binding residues that were extracted from the PLBSP Residue database for each ligand type.

**Table 1.** Numbers of ligand types and proteins and ligand-binding residues in the extracted dataset from the PLBSP Residue database

Ligand name	No. of ligand types	No. of ligand-binding proteins	No. of ligand-binding residues
Purine nucleotide	58	521	10,564
Lipid	117	224	4,737
Fe	2	130	1,005
Zn	2	576	5,128
Mn	1	230	1,772
FAD	1	123	4,168
AMP	1	54	1,013
SF4	1	71	1,392

**Fig. 4.**
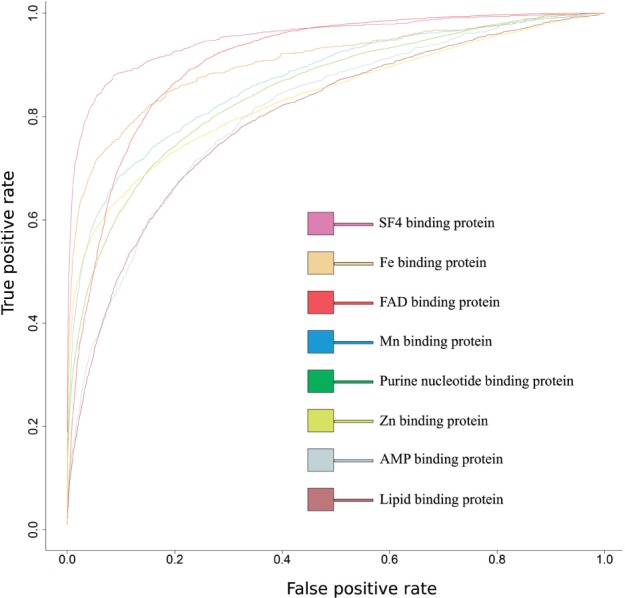
Predictive accuracy of predictors automatically generated using the pipeline workflow. Accuracies of the prediction tools are automatically calculated and are indicated as AUC values. The *x*-axis shows the false positive rate (FPR) and the *y*-axis shows the true positive rate (TPR). The ROC plot shows curves for the eight ligand-binding proteins listed in Tables 1 and 2

The performance of these predictors was plotted using receiver operating characteristic (ROC) curves (Table 2 and Fig. 4) and the accuracy of the prediction tools was evaluated using 5-fold cross validation (LeDell *et al.*, 2012; Sing *et al.*, 2005). These analyses indicated sufficient performance for practical use.

**Table 2.** Performances of SVM-based prediction tools generated for eight ligand-binding proteins in Table 1

Ligand name	Sens. (%)	Spec. (%)	MCC	AUC
Purine nucleotide	37.4	98.0	0.484	0.850
Lipid	24.0	97.4	0.331	0.798
Fe	49.3	99.3	0.615	0.904
Zn	40.6	99.2	0.555	0.835
Mn	34.7	99.1	0.484	0.869
FAD	43.2	96.8	0.630	0.906
AMP	20.8	98.3	0.320	0.808
SF4	75.5	97.7	0.781	0.952

LigandRFs uses a RF ensemble with PSSMs as features and presents the results using Matthews correlation coefficient (MCC) (Chen *et al.*, 2014). The authors trained their predictor using the CASP8 dataset and then tested it against the CASP9 dataset (López *et al.*, 2009; Schmidt *et al.*, 2011). They reported an MCC of 0.4 and sensitivity of 42.7%. For the metal binding proteins of the CASP9 dataset, their results were better, ranging from 0.2 (for a calcium binding protein) to 0.81 for two zinc binding proteins and 0.85 for a Fe (II) binding protein. The average MCC for CASP9 metal binding proteins was 0.55. Our predictor for iron using SVM achieved a MCC of 0.62. For zinc, the MCC was 0.56 (using SVM). Our predictors generally performed reasonably well when compared with the average case but the best case sensitivity was better for LigandRFs.

As for other types of classifier, a naive Bayesian classifier was used for 42 transmembrane protein sequences using a PSSM profile, the BLOSUM62 matrix, and various amino acid properties as features (Suresh *et al.*, 2015). Suresh gives their results in terms of accuracy and sensitivity, achieving 65.25% accuracy and 50.18% sensitivity. Our predictor achieves reasonably good sensitivity/specificity for some ligands, although average sensitivity is somewhat less (40.69%). The hidden Markov SVM method uses 1124 protein chains from PDB (Liu *et al.*, 2014). The authors divided the dataset into hetero-complex and homo-complex protein chains and obtained features using accessible surface area and order profile propensity (which are profile-based features) and a PSSM profile. When they tested the method using a PSSM profile and solvent-accessible surface area, they obtained an AUC of 0.825 and MCC of 0.455. Including order profile propensity helped them to improve the MCC to 0.474. This method compares quite favorably with our predictors in terms of MCC and AUC. Our predictors achieve similar AUC and MCC for many ligands, whereas sensitivity is lower than that of the HM-SVM method (74.0% against our 40.69%). However, our predictor performance remains similar as the study reported a significant hit in specificity (53.5% against our 98.6%).

We also compared our predictor with specific ligand-binding site predictors for metal-binding residues (Shu *et al.*, 2008). In one study, the authors attempted to predict zinc-binding sites using SVM and homology score. They used 2727 chains from PDB (with 235 zinc-binding chains). Using PSSM profile, conservation score and homology score as features, they obtained an average AUC of 0.723. Our predictor achieved an AUC of 0.835 using SVM, although the sensitivity was 40.6%. Another study (Lu *et al.*, 2012) showed metal cation-binding site prediction performance comparable to that in the present study. Details of the evaluation are described in the online methods. For lipid binding sites, a previous study (Xiong *et al.*, 2010) using a PSSM-based method achieved an AUC of 0.796, which is similar to the AUC obtained by our system.

Note that the above performance comparison with the existing methods was performed on different datasets: the performance values of the existing methods were taken from the original papers. So the performance improvement may be due to new datasets but not the prediction methods.

### 3.2 Optimization of SVM parameters

During the automatic generation of prediction tools, the system optimizes SVM parameters using GA within Pyevolve (Perone, 2009). In this study, we evaluated the performance of GA in optimizing iron cation binding site predictions using the criteria of prediction accuracy as reported by AUC as well as execution times. The evaluation included 5-fold cross-validation. The average AUC for each prediction tool was improved by changing parameters using GA. In this experiment, the AUC at the start of the GA run was 0.691 and the AUC obtained after 100 generations was 0.934 with a standard error of 0.47%, indicating that GA facilitates parameter tuning of SVM within realistic calculation times (Table 3).

**Table 3.** Average AUC and execution times of the generation of the GA as a parameter-tuning method for prediction of iron cation binding proteins

GA generation	Run time (s)	SVM param. cost	SVM param. sigma	Window size *w*	SVM AUC	Std error (AUC) (%)
0		17.78	1.99	17	0.691	1.76
1	1,753	0.54	1.26	5	0.827	1.22
2	2,134	29.42	1.29	9	0.799	1.36
3	2,217	29.42	0.37	9	0.881	1.04
4	2,255	13.60	2.79	9	0.741	1.57
5	2,333	13.60	1.83	9	0.766	1.42
10	2,584	25.23	0.32	9	0.894	0.94
20	3,122	25.23	0.32	9	0.894	0.94
30	3,643	25.23	0.32	9	0.894	0.94
40	3,964	25.23	0.32	9	0.894	0.94
50	4,271	25.23	0.32	9	0.894	0.94
60	4,674	25.23	0.32	9	0.894	0.94
70	5,013	25.23	0.32	9	0.894	0.94
80	5,389	25.23	0.32	9	0.894	0.94
90	5,507	17.84	0.32	9	0.894	0.94
100	5,960	17.84	0.15	9	0.934	0.47

Parameters for SVM affect to varying degrees how the algorithm finds the best separation between different classes. SVM uses a hyperplane to separate the points and calculate a value for the separation. This separation is maximized by changing the hyperplane. Separation can be calculated using Gaussian distance as used in our study or some other function such as Laplace or hyperbolic tangent. The calculations are performed by a kernel function and used by SVM to calculate a distance value between two set of feature vectors. The radial basis Gaussian kernel function can be written as

(1) $$K(x^{1},x^{2})=e^{{-\frac{∥x^{1}-x^{2}∥}{2\sigma^{2}}}^{2}}$$

where ***x***^1^ and ***x***^2^ are feature vectors.

The sigma parameter (σ) inversely affects the separation value returned by the kernel and in turn modifies how the machine learning algorithm learns. Smaller sigma values may improve data fitting, but very small values may lead to overfitting of data, as kernel values will approach zero.

The cost parameter is a regularization parameter to help control bias (underfitting) or overfitting. A large cost value will reduce the distance allowed between a point and hyperplane before the point is considered misclassified. Large cost values control bias, whereas low cost values control overfitting.

### 3.3 Performance comparison of the machine learning algorithms

Our system can use three machine learning algorithms: SVM, NN and RF (Bergmeir and Benitez, 2012; Kuhn, 2008). The performance of these algorithms is compared in Tables 4 and 5. Table 4 presents values obtained by a predictor optimized using grid search. Table 5 presents values from the predictor optimized using GA. Grid search can search a wider range of parameters and achieve higher accuracy than GA (Scrucca, 2013). Comparison between the tables shows how well GA performed for each predictor. GA is a heuristic method, so performance is expected to be lower. However, the predictors perform quite well when using GA, and average AUC dropped only by 4% for SVM, 6.7% for RF and 1% for NN.

**Table 4.** Average AUCs of three machine learning methods for grid search parameter optimization

Ligand name	Algorithm	Sensitivity (%)	MCC	AUC
Purine nucleotide	SVM	44.5	0.554	0.869
	NN	40.3	0.374	0.765
	RF	27.3	0.445	0.858
Lipid	SVM	45.7	0.516	0.863
	NN	42.6	0.338	0.753
	RF	24.8	0.418	0.851
Iron cation	SVM	61.2	0.718	0.940
	NN	59.9	0.641	0.894
	RF	46.9	0.635	0.940

A window size of nine was used for all cases. For SVM, sigma was 0.1 and cost was 1.0. For NN, the number of nodes was 25 and learning rate was 0.1. For RF, number of trees were 1501 and sampling size per tree was 20. The test dataset was 15% of the full dataset for each ligand. It was randomly sampled and removed from the dataset. The remaining dataset was used for training.

**Table 5.** Average AUCs of three machine learning methods for GA parameter optimization

Ligand name	Algorithm	Parameters	Sensitivity (%)	MCC	AUC
Purine nucleotide	SVM	Sigma = 0.16, Cost = 22.5, *w* = 5	41.3	0.213	0.834
	NN	#Nodes = 44, Learning rate = 3.26, *w* = 13	20.8	0.295	0.636
	RF	#Trees = 2023, #Iterations = 20, *w* = 17	25.9	0.450	0.849
Lipid	SVM	Sigma = 0.78, Cost = 25.27, *w* = 19	13.5	0.289	0.803
	NN	#Nodes = 49, Learning rate = 0.66, *w* = 9	44.6	0.381	0.775
	RF	#Trees = 1611, #Iterations = 3, *w* = 11	19.5	0.387	0.854
Iron cation	SVM	Sigma = 0.32, Cost = 17.66, *w* = 3	45.6	0.654	0.911
	NN	#Nodes = 46, Learning rate = 3.26, *w* = 9	32.0	0.458	0.803
	RF	#Trees = 991, #Iterations = 29, *w* = 9	35.0	0.579	0.943

The best parameters found are as this table. The test dataset was 15% of the full dataset for each ligand and was same as used in Table 4.

Whether using GA or grid search, we reach the same conclusion as other studies: that SVM is one of the best-performing machine learning tools available for binding-site residue prediction. RF also gives promising results, but sensitivity suffers. NN based on a single hidden layer with a maximum of 25 nodes performed reasonably well. Better results may be expected by adding layers to the NN or changing the prediction function for RF. However, among off-the-shelf machine learning algorithms, SVM is the best method, while RF and NN are sufficiently different and accurate to be useful alternative options to SVM.

## 4 Conclusion

We present a user-friendly automatic production pipeline for protein–ligand-binding site predictive tools using multi-omics big data. The pipeline’s core data is organized by ontology as an evidence. Implementation of this system in a pipeline of Semantic Web technology-based Web tools allow users to specify a ligand and receive the tool within 0.5–1 day. We have demonstrated its high prediction accuracy for several ligands. The present pipeline system has backend-linked open data comprising ∼30 billion triples as an RDF graph. It is available as a Web application that can be accessed and used at URL http://utprot.net, UTProt Galaxy. We evaluated the software by automatically generating binding-site predictors for eight types of ligand using SVM and GA-based parameter optimization. We also evaluated performance for three types of ligand and three machine learning algorithms: SVM, RF and NNs, and two parameter optimization methods: GA-based and grid search. We are now developing methods to extract only biologically relevant ligands; in this study we excluded only ligands that are apparently buffer media used for crystallization. A general method that accommodates similarity among ligands should be designed. Our reasoning search of ligands based on ontology hierarchies partially addresses this need, but more general ligand grouping based on stereochemistry or minor substituents can be conceived. The combination of our system with homology-based or template-based methods is also a subject of future work.

## Supplementary Material

Supplementary Data
