# Supplementary material for: Automatic generation of bioinformatics tools for predicting protein–ligand binding sites
Source: Bioinformatics. 2015 Nov 5;32(6):901–7. doi: 10.1093/bioinformatics/btv593 (PMC4803387; doi:10.1093/bioinformatics/btv593)
Supplement: Supplementary Data [file supp_32_6_901__index.html]

Automatic generation of bioinformatics tools for predicting protein–ligand binding sites — Supplementary Data 

# Automatic generation of bioinformatics tools for predicting protein–ligand binding sites

## Supplementary Data

files

- Supplementary Data - pdf file
