# Supplementary material for: Automatic generation of bioinformatics tools for predicting protein–ligand binding sites
Source: Bioinformatics. 2015 Nov 5;32(6):901–7. doi: 10.1093/bioinformatics/btv593 (PMC4803387; doi:10.1093/bioinformatics/btv593)
Supplement: Supplementary Data [file supp_btv593_Supplementary-Note-S1-S2.pdf]

Lecture Note

Automatic generation of bioinformatics tools for protein–ligand binding site prediction

Yusuke Komiyama, Masaki Banno, Kokoro Ueki, Gul Saad, and Kentaro Shimizu

(The University of Tokyo)

Background: Prediction and Identification of Ligand Binding Sites

Experimental Methods

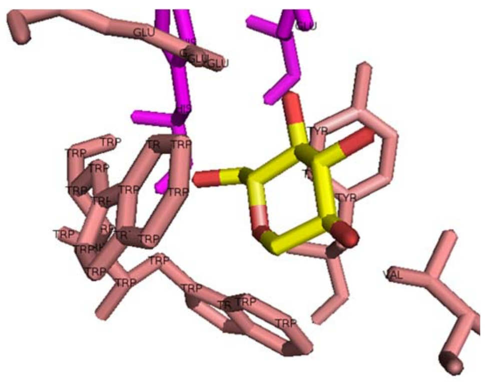

- Biochemical experiments
- Experimental structure determination

etc. …

Computational Methods

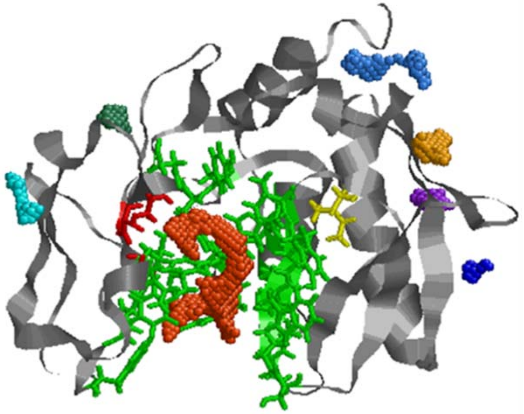

- Binding-site prediction
- Docking simulation

- **Experimental methods require much more time and greater cost.**
- **Docking simulations can only be applied to known structures.**

Sequence-based ligand binding site prediction

- Sequence-based ligand binding site prediction is useful.
- It does not take much time and is inexpensive.
  - It can be applied to genome-wide analyses.
- Many existing methods use machine-learning techniques.
- However, there are many kinds of ligands that have no binding site prediction tools.
- In this case, a user should develop a new binding site prediction tool.

Developing a new ligand binding site prediction tool require considerable time and cost.

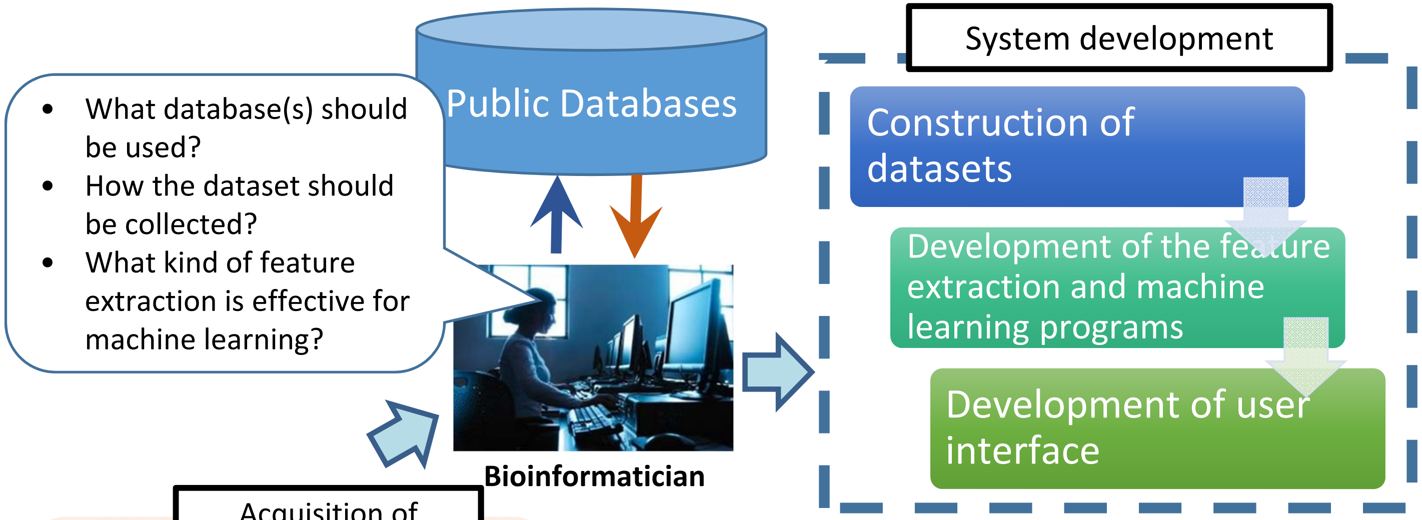

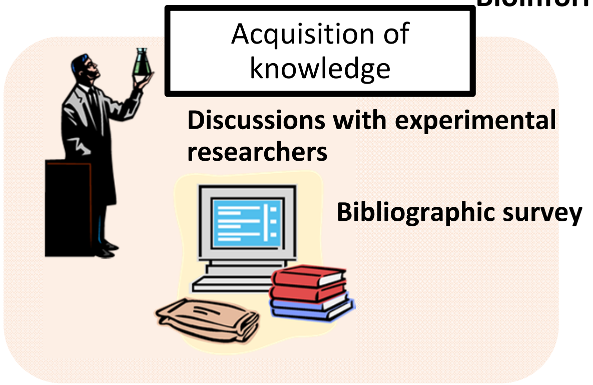

- Ligand binding site prediction tools should be developed for individual ligands.
- However, there are common steps for developing ligand binding site prediction tools .

Research purpose

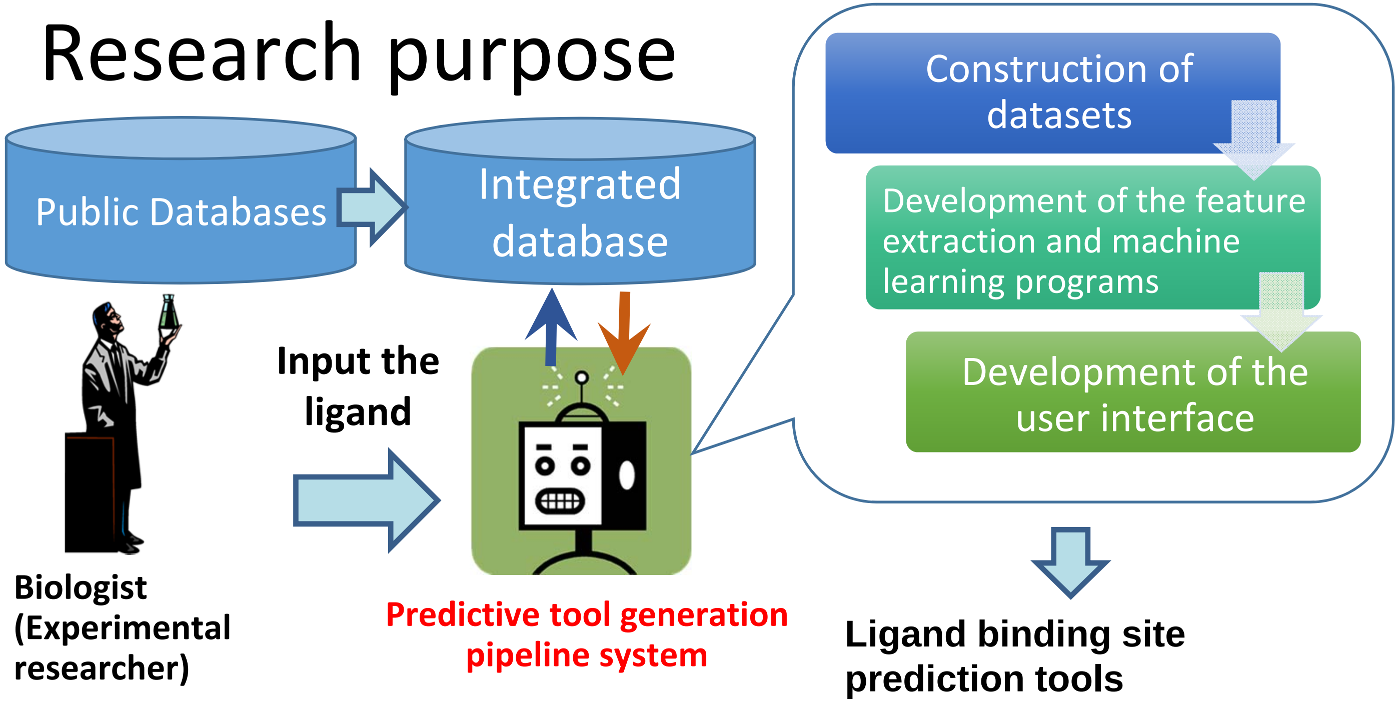

Developed a system for generating protein–ligand binding prediction tools automatically.
This system is implemented as a pipeline of Web tools based on the Semantic Web technique.

Advantages of our system

- **A user has only to specify a ligand and can get the prediction tool within half to one day.**
- Users can generate a prediction tool for many kinds of ligand as they are needed (on demand).
- User-interface is uniform and easy to use.
- Re-use of prediction tools can be promoted.

Method : Overview of our system

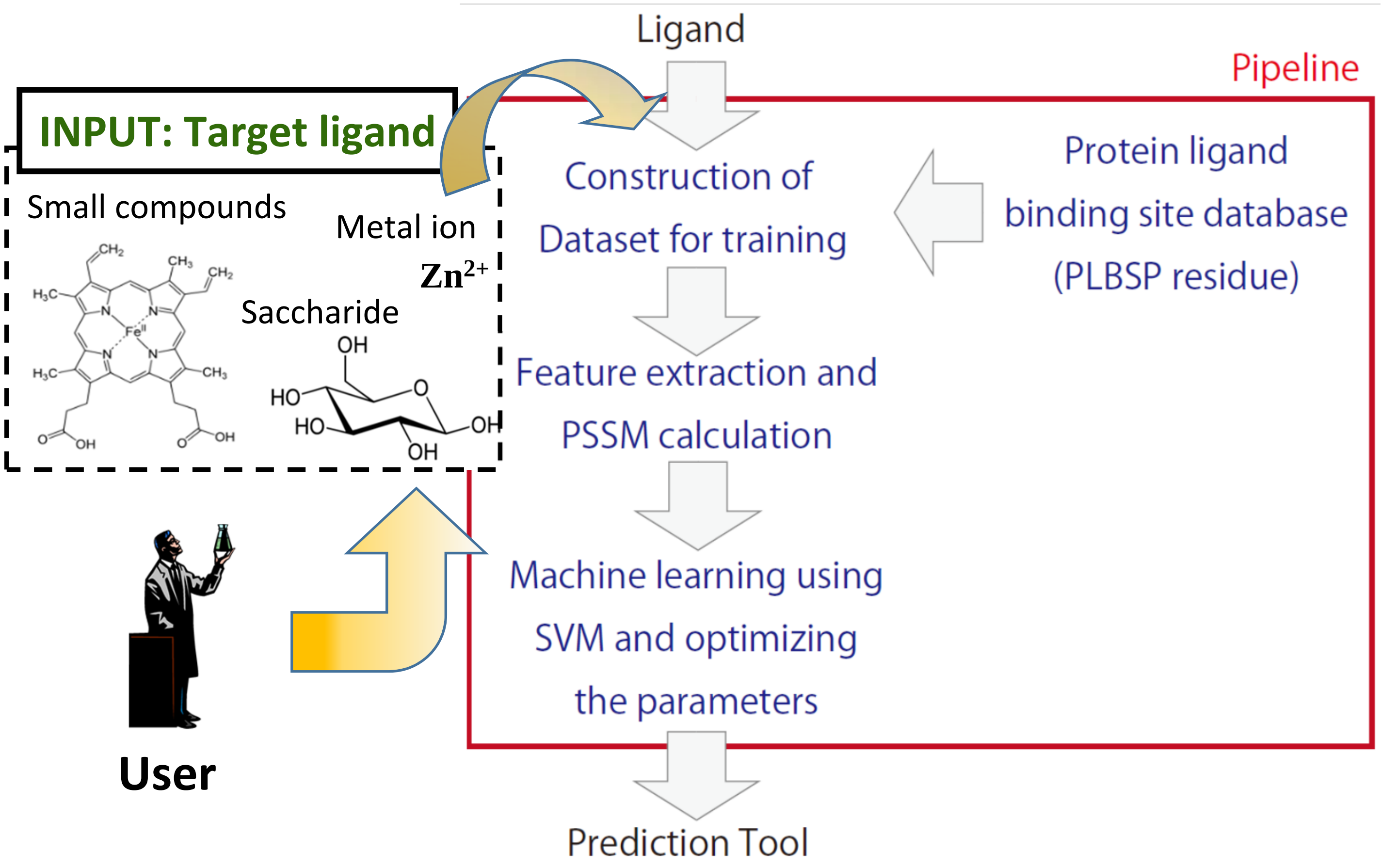

Method : Flow of prediction

INPUT: Amino acid sequence

…SERDFLALALGGT…

Feature extraction and generation of PSSM

Support vector machine

Automatic parameter tuning

OUTPUT: Ligand binding residue

…SERDFLALDLGGT…

Predicted ligand binding residue

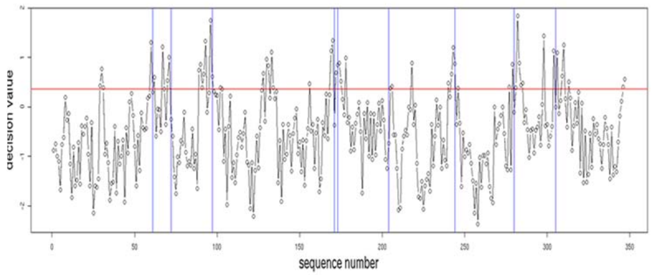

Decision values are used for determining binding site residue or not.

Contents of our research

Development of an integrated database – PLBSP Residue

- RDFize binding residue information of PDB
- RDFize covalent bond information between ligands
- RDFize cross reference between PDB and UniProtKB

Development of dataset generator

- Generate positive and negative datasets from the databases.
- Remove the sequence redundancies
- Calculate PSSM

Development of prediction tool generator pipeline

- Convert to feature vector
- Perform prediction using SVM
- Parameter optimization of SVM

Roll-out of the system

- Start up of UTProt Galaxy
- Development of the workflow for generating ligand binding site prediction tools
- Start up a backend of the generated tools manager, UTprot CKAN

PLBSP Residue

- Protein Ligand Binding Site Pair Residue (PLBSP Residue) is a RDFized graph database of ligand-binding residues
- This database contains the protein–ligand interaction sites available in Protein Data Bank (PDB)
- Interaction site residues are defined as residues that contain at least one atom within n Å of any ligand atom in an experimentally determined structure
- Efficient structure comparison and analysis are enabled by the Octree data structure
- We RDFized EBI SIFTS, which curates the relationships between UniProtKB and PDB residues, and incorporated this into PLBSP Residue

Outline of the RDF schema of PLBSP Residue

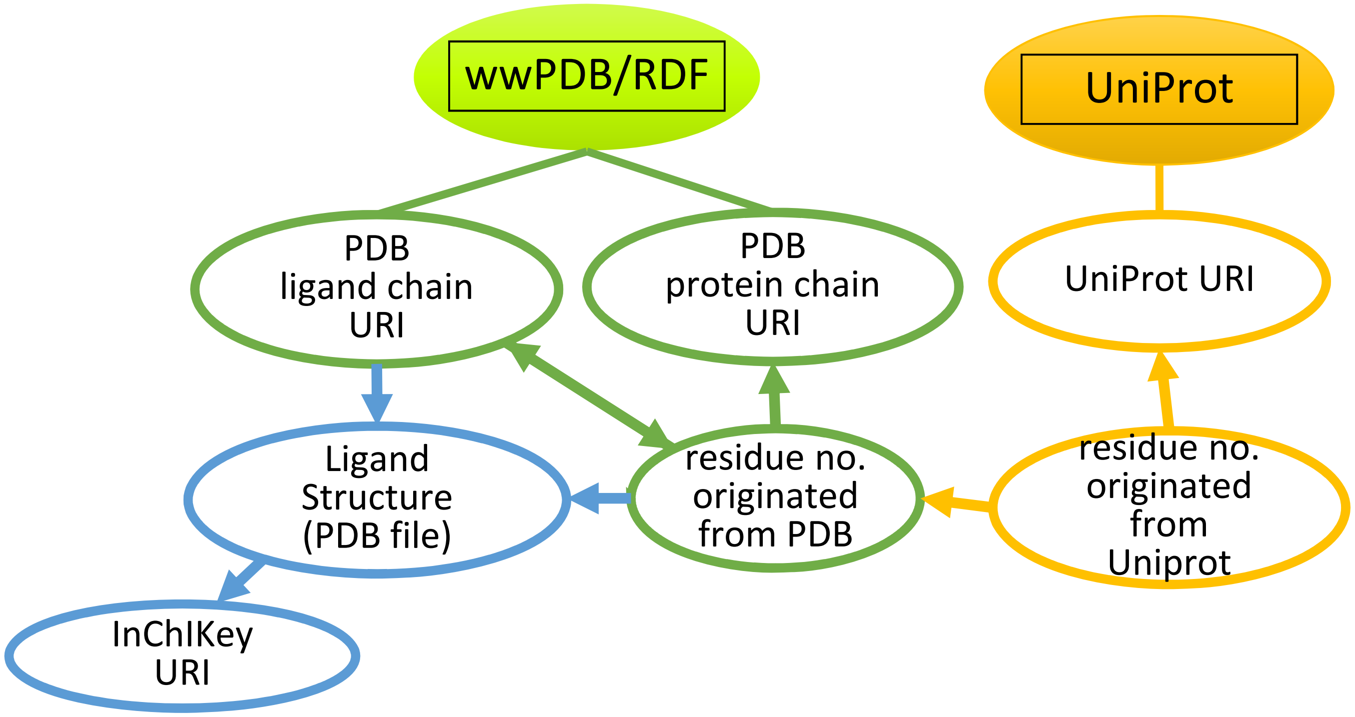

Users specify the HETATM code of a ligand to search for proteins that bind the ligand. The search options include the resolution, Gene Ontology, and protein families.

Advantages of mapping to UniProt

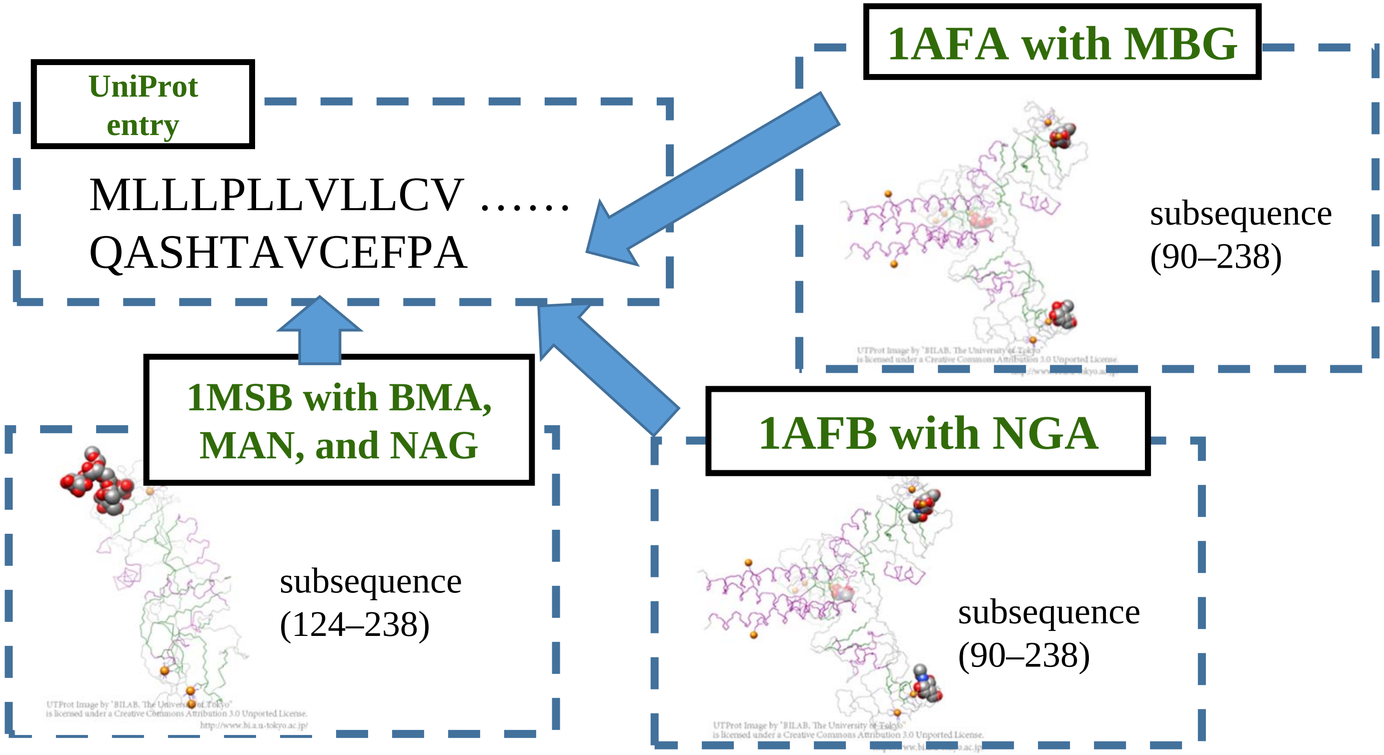

- Multiple PDB entries are linked to a sequence.
- Full sequences can be obtained.
- Other information linked to UniProt can be referenced.

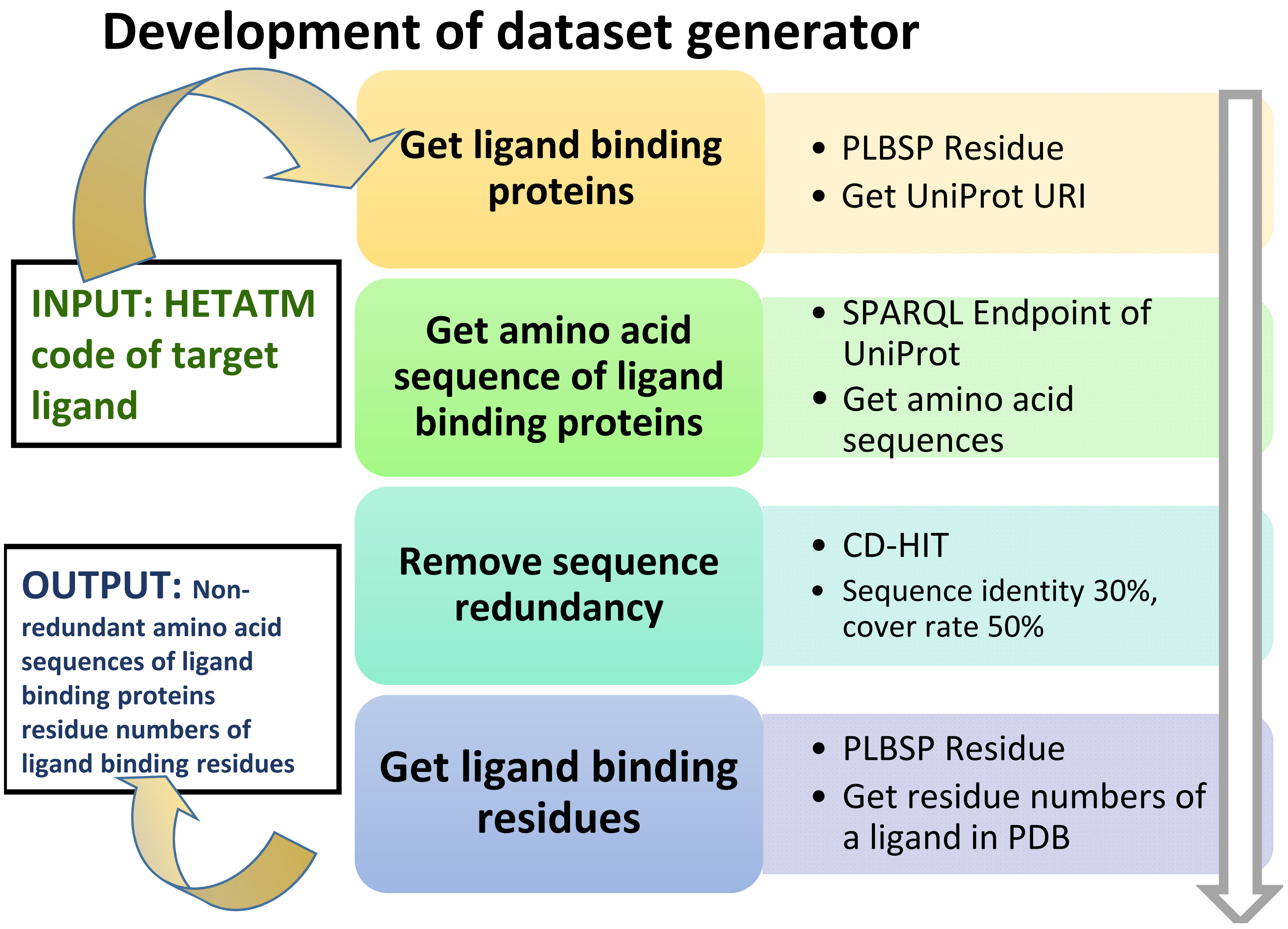
Development of dataset generator
INPUT: HETATM code of target ligand
Get ligand binding proteins
PLBSP Residue
Get UniProt URI
Get amino acid sequence of ligand binding proteins
SPARQL Endpoint of UniProt
Get amino acid sequences
Remove sequence redundancy
CD-HIT
Sequence identity 30%, cover rate 50%
Get ligand binding residues
PLBSP Residue
Get residue numbers of a ligand in PDB
OUTPUT: Non-redundant amino acid sequences of ligand binding proteins residue numbers of ligand binding residues

Prediction tool generator pipeline

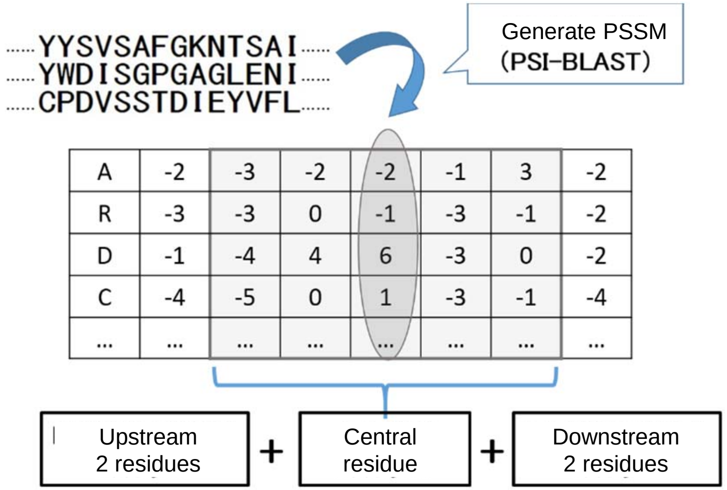

Generate PSSM from multiple sequence alignment by using PSI-BLAST.
Use *w* residues around the central residue as the input feature of SVM

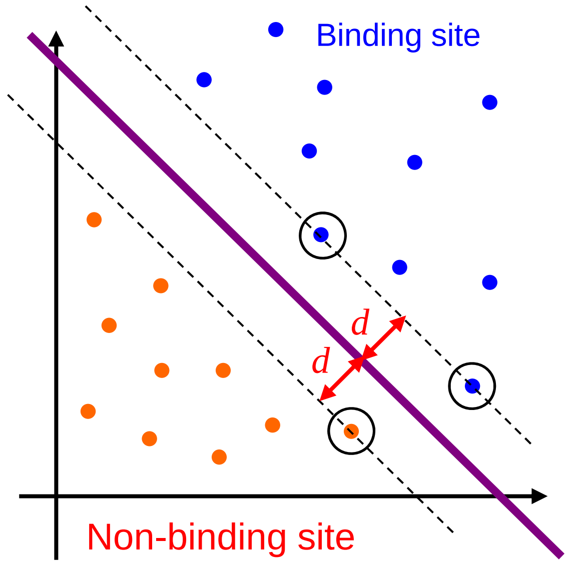

Collect positive dataset from the ligand binding residues and negative dataset from the residues 5–25 distant from the ligand binding residues. SVM is trained by these datasets. Parameter optimization is performed by using a genetic algorithm.

Roll-out of the system – UTProt Galaxy

Press “Users” menu and select “Registration” for user registration

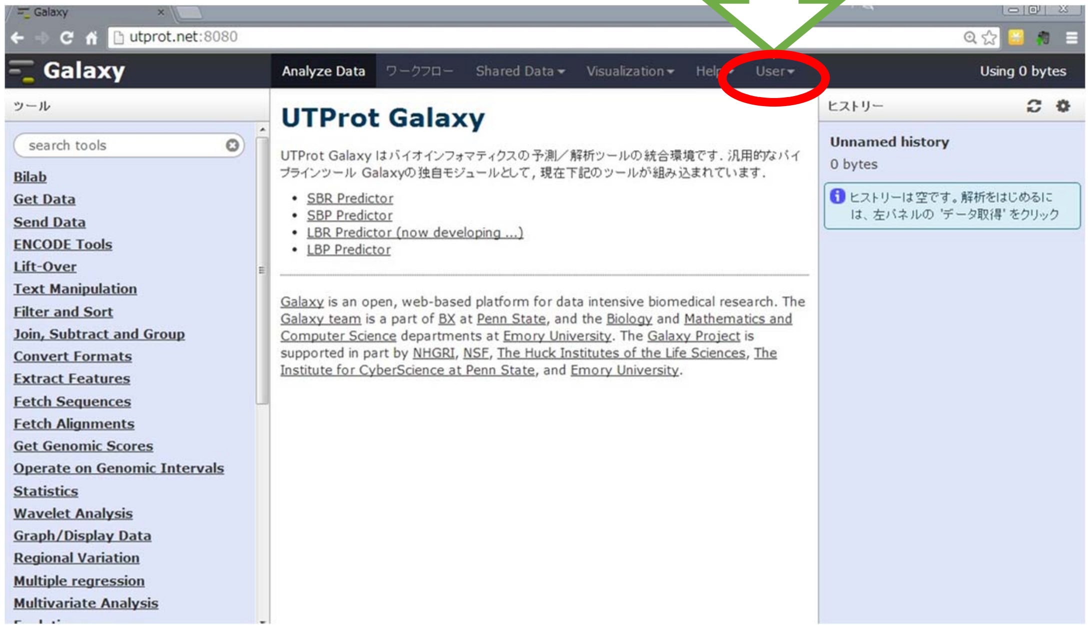

After registration to UTProt Galaxy, a prototype of our workflow can be used by selecting “Shared Data” -> “Published Workflows”.

Number of ligand types, proteins, and ligand-binding residues in the dataset extracted from the PLBSP residue database

Ligand Name	No. of Ligand Types	No. of Ligand-Binding Proteins	No. of Ligand-Binding Residues
Purine nucleotide	58	521	10564
Lipid	117	224	4737
Fe	2	130	1005
Zn	2	576	5128
Mn	1	230	1772
FAD	1	123	4168
AMP	1	54	1013
SF4	1	71	1392

Performance of prediction tools generated

Ligand Name	Sens. (%)	Spec. (%)	MCC	AUC
Purine nucleotide	37.4	98.0	0.484	0.850
Lipid	24.0	97.4	0.331	0.798
Fe	49.3	99.3	0.615	0.904
Zn	40.6	99.2	0.555	0.835
Mn	34.7	99.1	0.484	0.869
FAD	43.2	96.8	0.630	0.906
AMP	20.8	98.3	0.320	0.808
SF4	75.5	97.7	0.781	0.952

Performances of prediction tools generated (AUC values)

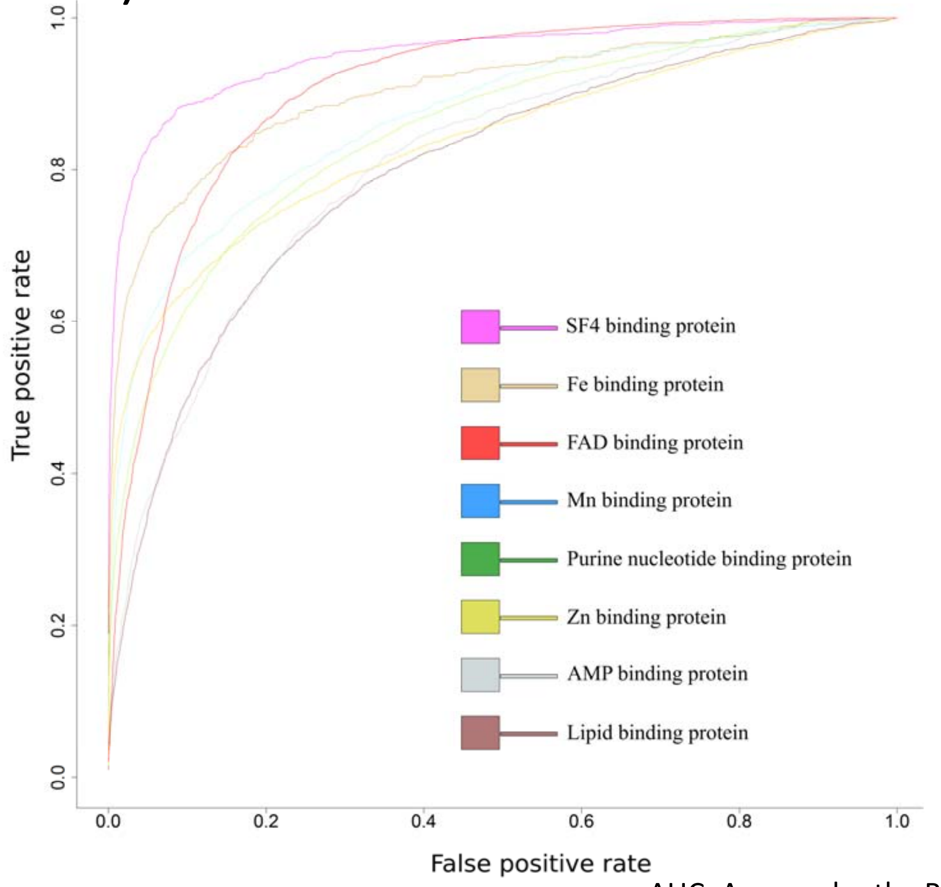

AUC: Area under the ROC curve

Improvements of parameters using the genetic algorithm

GA generation	Run time (sec.)	SVM param. cost	SVM param. sigma	Window size w	SVM AUC	Std error (AUC) (%)
**0**		17.78	1.99	17	0.691	1.76
**1**	1753	0.54	1.26	5	0.827	1.22
**2**	2134	29.42	1.29	9	0.799	1.36
**3**	2217	29.42	0.37	9	0.881	1.04
**4**	2255	13.60	2.79	9	0.741	1.57
**5**	2333	13.60	1.83	9	0.766	1.42
**10**	2584	25.23	0.32	9	0.894	0.94
**20**	3122	25.23	0.32	9	0.894	0.94
**30**	3643	25.23	0.32	9	0.894	0.94
**40**	3964	25.23	0.32	9	0.894	0.94
**50**	4271	25.23	0.32	9	0.894	0.94
**60**	4674	25.23	0.32	9	0.894	0.94
**70**	5013	25.23	0.32	9	0.894	0.94
**80**	5389	25.23	0.32	9	0.894	0.94
**90**	5507	17.84	0.32	9	0.894	0.94
**100**	5960	17.84	0.15	9	0.934	0.47

Performance comparison of the machine learning algorithms

Grid search parameter optimization

Ligand name	Algorithm	Sensitivity (%)	MCC	AUC
Purine nucleotide	SVM	44.5	0.554	0.869
	NN	40.3	0.374	0.765
	RF	27.3	0.445	0.858
Lipid	SVM	45.7	0.516	0.863
	NN	42.6	0.338	0.753
	RF	24.8	0.418	0.851
Iron cation	SVM	61.2	0.718	0.940
	NN	59.9	0.641	0.894
	RF	46.9	0.635	0.940

Performance comparison of the machine learning algorithms

Genetic algorithm parameter optimization

Ligand Name	Algorithm	Parameters	Sensitivity (%)	MCC	AUC
Purine nucleotide	SVM	Sigma = 0.16, Cost = 22.5, w = 5	41.3	0.213	0.834
	NN	#Nodes = 44, Learning rate = 3.26, w = 13	20.8	0.295	0.636
	RF	#Trees = 2023, #Iterations = 20, w = 17	25.9	0.450	0.849
Lipid	SVM	Sigma = 0.78, Cost = 25.27, w = 19	13.5	0.289	0.803
	NN	#Nodes = 49, Learning rate = 0.66, w = 9	44.6	0.381	0.775
	RF	#Trees = 1611, #Iterations = 3, w = 11	19.5	0.387	0.854
Iron cation	SVM	Sigma=0.32, Cost=17.66, w = 3	45.6	0.654	0.911
	NN	#Nodes = 46, Learning rate = 3.26, w = 9	32.0	0.458	0.803
	RF	#Trees = 991, #Iterations = 29, w = 9	35.0	0.579	0.943

Conclusions

- We have developed a system for generating protein–ligand binding prediction tools automatically.
- The performance is comparable to existing tools that were developed for individual ligands.
    - By using the generated tools, we can develop more elaborate systems.
- Our pipeline system can be applied to many kinds of ligands.

Future Work

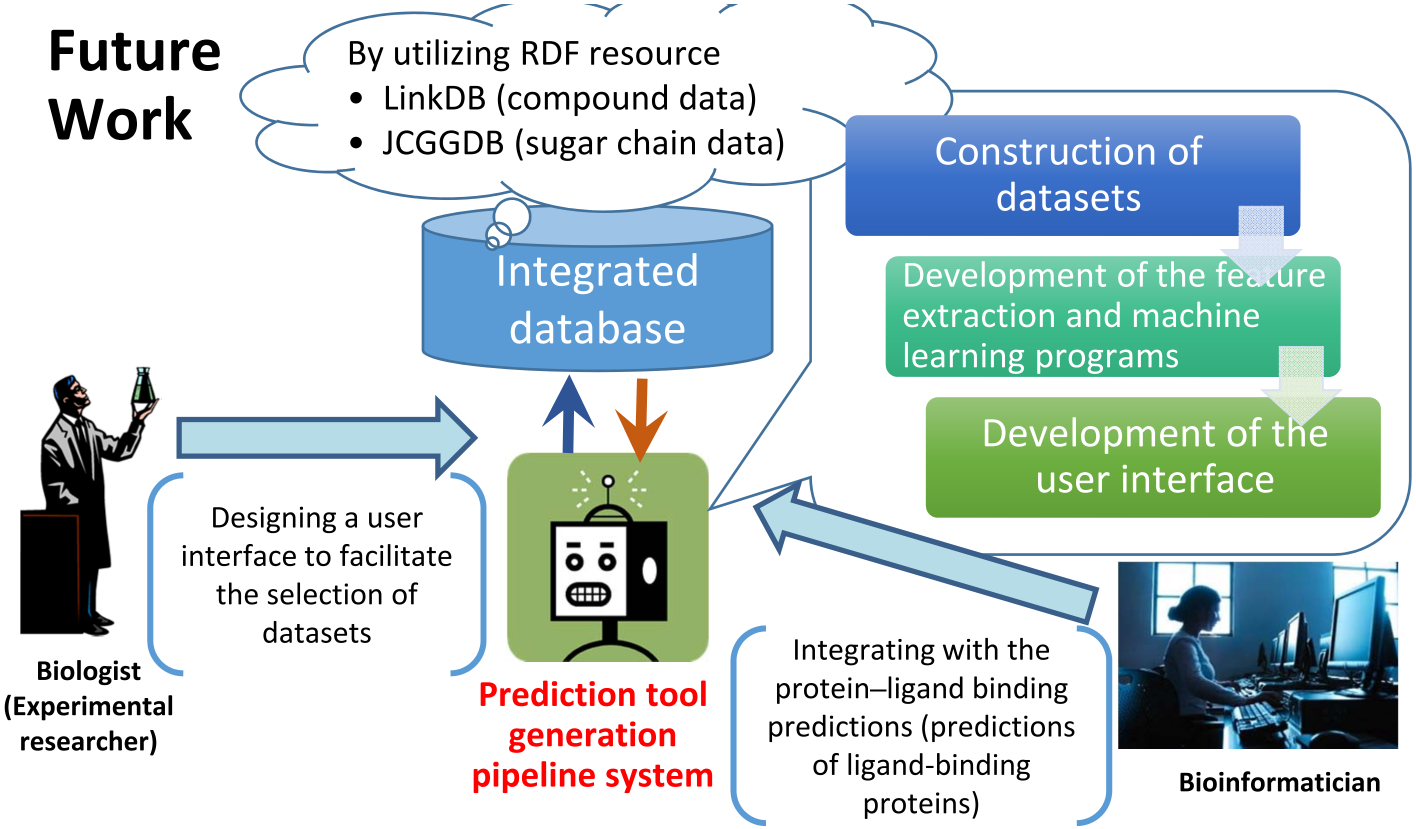

- Development of various prediction tools by applying our methods.
- Improve accuracy by incorporating other prediction methods and databases.
- Facilitate automatic reuse and customization.

Supplementary Note S2 Sample SPARQL queries in UTProt Galaxy pipeline.

Here, we describe the SPARQL queries in the pipeline. The query of "get a ligand binding proteins from multiple HETATM code" describes a next code in SPARQL in the "GLC (ALPHA-D-GLUCOSE)" case. In the query in a pipeline's code, those concrete ligand names are transposed to the variable.

```
PREFIX pdbo:<http://rdf.wwpdb.org/schema/pdbx-v40.owl#>
PREFIX dcterms:<http://purl.org/dc/terms/>
PREFIX edam:<http://edamontology.org/>
PREFIX sio:<http://semanticscience.org/resource/>
PREFIX up:<http://purl.uniprot.org/core/>
PREFIX obo:<http://purl.obolibrary.org/obo/>
SELECT DISTINCT ?uniprot WHERE {
  SERVICE <http://utprot.net:8890/sparql> {
    SELECT  ?uniprot WHERE {
      GRAPH <http://utprot.net/plbsp_residue> {
        ?het_res pdbo:atom_site.chem_comp.id "GLC".
        ?het_asym  dcterms:isPartOf ?het_res;
          rdf:type sio:SIO_010432;
          rdfs:seeAlso ?pdb_res.
        ?pdb_res rdf:type edam:data_1756;
          rdfs:seeAlso ?unpres.
        ?unpres rdf:type edam:data_1756;
          dcterms:isPartOf ?uniprot.
        MINUS { ?struct dcterms:hasPart ?het_asym; rdf:type obo:MOD_00000. }}}}}
```

Next one shows the query of "get binding residue from HETATM ID list and UniProtID list" in the "BOG (B-OCTYLGLUCOSIDE)" and "Q42187" case.

```
PREFIX pdbo:<http://rdf.wwpdb.org/schema/pdbx-v40.owl#>
PREFIX dcterms:<http://purl.org/dc/terms/>
PREFIX edam:<http://edamontology.org/>
PREFIX sio:<http://semanticscience.org/resource/>
PREFIX obo:<http://purl.obolibrary.org/obo/>
SELECT ?uniprot ?sqno WHERE {
  SERVICE <http://utprot.net:8890/sparql> {
    SELECT ?uniprot ?sqno WHERE {
      GRAPH <http://utprot.net/plbsp_residue> {
          ?het_res pdbo:atom_site.chem_comp.id ?hetid.
          ?het_asym  dcterms:isPartOf ?het_res;
            rdf:type sio:SIO_010432;
            rdfs:seeAlso ?pdb_res.
          ?pdb_res rdf:type edam:data_1756;
            rdfs:seeAlso ?unpres.
          ?unpres rdf:type edam:data_1756;
            dcterms:isPartOf ?uniprot;
            rdfs:label ?sqno.
          MINUS { ?struct dcterms:hasPart ?het_asym; rdf:type obo:MOD_00000. }

          VALUES ( ?uniprot ) {
            (<http://purl.uniprot.org/uniprot/O42187>)}
          VALUES ( ?hetid ) {
            ("BOG" ) }}
    } ORDER BY ?uniprot ?sqno}}
```

Last one shows the query of "get HETATM code from ChEBI class." in the "CHEBI_16646 (carbohydrate)" case.

```
PREFIX pdbo:<http://rdf.wwpdb.org/schema/pdbx-v40.owl#>
PREFIX dcterms:<http://purl.org/dc/terms/>
PREFIX obo:<http://purl.obolibrary.org/obo/>
SELECT DISTINCT * WHERE {
  SERVICE <http://utprot.net:8890/sparql> {
    SELECT DISTINCT *  WHERE {
      GRAPH <http://utprot.net/plbsp_residue> {
        ?chem_comp pdbo:link_to_chem_comp ?cc .
        ?cc rdf:type ?chebi  . }}}

  SERVICE <http://utprot.net:8890/sparql> {
    SELECT DISTINCT *  WHERE {
      GRAPH <http://utprot.net/chebi> {
        ?chebi rdfs:subClassOf ?superior_class1 ;
               rdf:type owl:Class ;
               rdfs:label ?name .
        ?superior_class1 rdfs:subClassOf ?superior_class2 ;
                         rdfs:label ?class_name1 ;
                         rdf:type owl:Class .
        ?superior_class2 rdfs:subClassOf obo:CHEBI_16646 ;
                         rdfs:label ?class_name2 ;
                         rdf:type owl:Class . }}}}
```

These SPARQL 1.1 queries can request a response to remote (another domain) database with SERVICE function, but these sample use local database. Besides, graph database has semantic reasoning function usually using comparative multi graphs.
